# Prognostic Value of Combined Neutrophil-to-Lymphocyte Ratio and Imaging Tumor Capsule in Solitary Hepatocellular Carcinoma Patients after Narrow-Margin Hepatectomy

**DOI:** 10.3390/jcm13020351

**Published:** 2024-01-08

**Authors:** Desheng Chen, Pengjuan Mao, Chen Sun, Xuhui Fan, Qi Zhu, Zeping Chen, Zeping He, Yichao Lou, Hongcheng Sun

**Affiliations:** 1 Department of General Surgery, Shanghai General Hospital, Shanghai Jiao Tong University School of Medicine, Shanghai 200080, China; cdsdoctor@sjtu.edu.cn (D.C.); zhuqiwish@sina.com (Q.Z.); chenzp99@sjtu.edu.cn (Z.C.); hezeping19980913@163.com (Z.H.); cxzxlych@126.com (Y.L.); 2 Department of Clinical Pharmacy, Shanghai General Hospital, Shanghai Jiao Tong University School of Medicine, Shanghai 200080, China; mao_0701@sjtu.edu.cn; 3 Clinical Research Center, Shanghai General Hospital, Shanghai Jiao Tong University School of Medicine, Shanghai 200080, China; sc0109@sjtu.edu.cn; 4 Department of Radiology, Shanghai General Hospital, Shanghai Jiao Tong University School of Medicine, Shanghai 200080, China; fanxuhui@sjtu.edu.cn

**Keywords:** solitary hepatocellular carcinoma, narrow-margin hepatectomy, neutrophil-to-lymphocyte ratio, imaging tumor capsule, prognosis

## Abstract

Background: The study aimed to investigate the clinical value and prognostic patterns of the neutrophil-to-lymphocyte ratio (NLR) and imaging tumor capsule (ITC) in solitary hepatocellular carcinoma (HCC) patients undergoing narrow-margin hepatectomy. Methods: Data for solitary HCC patients treated with narrow-margin surgery were extracted from Shanghai General Hospital. Clinical features of recurrence-free survival (RFS), overall survival (OS), and early recurrence were investigated by Cox/logistic regression. The significant variables were subsequently incorporated into the nomogram pattern. Survival analysis stratified by NLR and ITC was also performed. Results: The study included a cohort of 222 patients, with median RFS and OS of 24.083 and 32.283 months, respectively. Both an NLR ≥ 2.80 and incomplete ITC had a significant impact on prognosis. NLR and ITC independently affected RFS and OS, whereas alpha-fetoprotein (AFP) and ITC were identified as independent factors for early relapse. The RFS and OS nomogram, generated based on the Cox model, demonstrated good performance in validation. The combination of NLR and ITC showed greater predictive accuracy for 5-year RFS and OS. Subgroups with an NLR ≥ 2.80 and incomplete ITC had the worst prognosis. Conclusions: Both NLR and ITC significantly affected RFS, OS, and early recurrence among solitary HCC patients who underwent narrow-margin hepatectomy. The combination of NLR and ITC has the potential to guide rational clinical treatment and determine the prognosis.

## 1. Introduction

Primary liver cancer, predominantly hepatocellular carcinoma (HCC), remains the leading cause of cancer-related mortality globally [1]. While significant improvements in treatment strategies, such as the synergistic anticancer strategy of locoregional therapies and immunomodulators, have been made in recent decades, the dismal prognosis of patients with HCC, particularly those without available surgical options, has not been eliminated [2,3]. Even if HCC is treatable by curative hepatectomy, the postoperative recurrence rate after initial surgery is as high as 70% [4]. Given the remaining liver tissue and the complexity of the hepatic vascular system, surgical resection with narrow margins (≤1 cm) is the only curative option when feasible [5,6]. Consequently, a significant proportion of patients undergo narrow-margin hepatectomy, for which postoperative recurrence is an evident concern [7]. HCC is renowned for its heterogeneity, and the great variation in patients complicates clinical individualized management, resulting in limitations in predicting the prognosis and monitoring the recurrence of HCC, making it impossible to optimize treatment regimens promptly [3,8]. However, little research has been conducted on the patterns of HCC recurrence, particularly in patients who have undergone curative surgery with a narrow margin.

A tumor capsule (TC) has been regarded as a symptom associated with the advancement of HCC [9]. Conversely, alternative research has demonstrated that a TC is an innately protective mechanism that inhibits hepatocarcinogenesis. For instance, complete imaging TC (ITC) is an independent predictor of improved prognosis [10]. Nevertheless, there are still ongoing debates regarding the impact of TC on prognosis in HCC, and its predictive power requires further investigation. TC formation is caused by a fibrotic reaction in the tumor microenvironment (TME) [11]. Notably, fibrosis significantly drives the progression of HCC due to sustained inflammatory stimulation, which is a key mechanism contributing to hepatocarcinogenesis [12]. In addition, the neutrophil-to-lymphocyte ratio (NLR), the most prominent inflammatory marker, has been suggested as an indicator of the cancer-related inflammation status in HCC [13]. HCC cases characterized by an elevated NLR tend to have an adverse prognosis [14,15]. Collectively, an integrated ITC and NLR may enhance the efficacy of a predictive nomogram for HCC patients undergoing curative surgery with a narrow margin.

The objective of this research was identifying potential prognostic factors, particularly recurrence, for patients with solitary HCC after narrow-margin hepatectomy. In addition, we determined if the combination of ITC and NLR could predict clinical outcome, encompassing early recurrence and recurrence-free survival (RFS) as well as overall survival (OS) for these patients.

## 2. Methods

### 2.1. Research Objects

**Figure 1:**
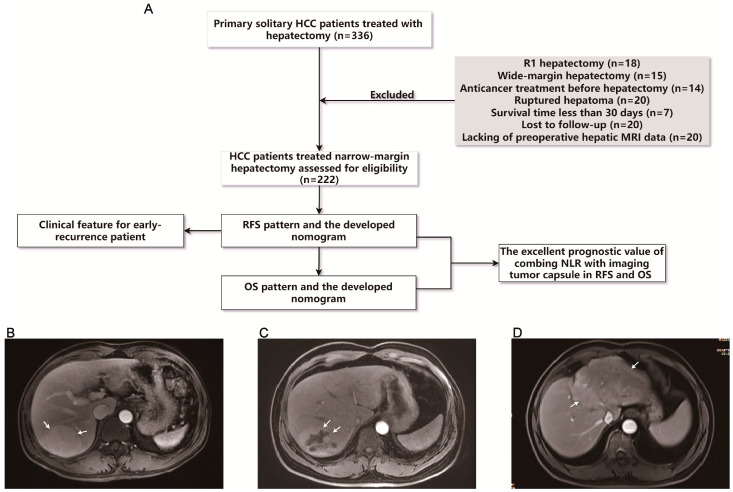
Flowchart and MRI features. (**A**) Flowchart portraying this study. Representative MRI images of (**B**) complete ITC and (**C**,**D**) incomplete ITC. The gray box illustrates the exclusion criteria. The arrows indicate the tumor. HCC: hepatocellular carcinoma; MRI: magnetic resonance imaging; RFS: recurrence-free survival; OS: overall survival; NLR: neutrophil-to-lymphocyte ratio; ITC: imaging tumor capsule.

Between January 2013 and July 2019, a cohort of 336 patients with a diagnosis of primary solitary HCC was included in this study at the Shanghai General Hospital in Shanghai, China. The ethics committee of our center granted approval (Approval No. 2020K038) for this study. Criteria for participant exclusion were depicted as follows: (1) R1 hepatectomy, (2) wide-margin hepatectomy, (3) anticancer treatment before hepatectomy, (4) ruptured HCC, (5) survival time less than 30 days, (6) lost to follow-up, and (7) missing preoperative hepatic magnetic resonance imaging (MRI) data (Figure 1A). Written informed consent was received from all patients involved, allowing for the utilization of their clinical data.

### 2.2. Data Collection

The clinical data utilized in this study were sourced from the medical records of Shanghai General Hospital. Characteristics included patient demographic information, preoperative laboratory values, preoperative MRI findings, and pathologic reports. The NLR at baseline was calculated by dividing the count of neutrophil by the count of lymphocyte. One week prior to surgery, the enhanced signal MRI was conducted. Two professional radiologists independently evaluated the features of the MRI images without prior clinical knowledge. The ITC was classified as either completely encapsulated (Figure 1B) or incompletely encapsulated (disrupted capsule or absence of capsule) (Figure 1C,D). The pathologists provided a report on the resection margin width, which represents the closest distance from the edge of the lesion to the transection margin of the parenchyma. Specifically, resected margins less than or equal to 10 mm were considered narrow, and vice versa.

### 2.3. Post-Treatment Monitoring

As per our hospital’s standard protocol, our outpatient clinic ensured that all patients received postoperative follow-up care, which was administered every three months for the initial two-year period after surgery and every six months thereafter [16]. The surveillance of tumor recurrence was conducted by the utilization of abdominal ultrasound and computed tomography (CT)/MRI imaging, along with tumor markers, including alpha-fetoprotein (AFP) and des-γ-carboxy prothrombin (DCP). HCC recurrence was characterized as the emergence of a newly detected tumor lesion on imaging studies, regardless of whether AFP or DCP levels were elevated. Moreover, progressive tumor marker elevation alone was also considered a recurrence. The ultimate follow-up date was 1 July 2020. HCC recurrence occurring within 2 years of hepatectomy was classified as early recurrence, while the time interval between hepatic resection and HCC recurrence or the last follow-up was considered as RFS. Regarding OS, the duration between hepatic resection and death or the conclusion of follow-up was determined.

### 2.4. Statistical Analysis

The R software (version 4.2.1) was utilized to analyze graphical displays and data. Continuous variables were expressed as median and categorical variables as count, as appropriate. The Mann–Whitney U test, chi-squared test, or Yates’ correction, as appropriate, were employed to compare baseline characteristics between the two groups. Using Kaplan–Meier (K-M) curves, survival was analyzed. Cox regression or logistic analysis was used to identify distinct risk factors. A nomogram integrating independent parameters in the Cox model was constructed and evaluated using K-M curves, decision curve analysis (DCA), and a calibration plot. A *p*-value below 0.05 denoted a statistically significant difference (* *p* < 0.05).

## 3. Results

### 3.1. Clinicopathologic Baseline between Patients with and without Recurrence

From the initial 336 consecutive patients, the final analysis included 222 patients with primary solitary HCC who underwent R0 resection with narrow margin (Figure 1A). The overall cohort had a median OS of 32.283 months and a median RFS of 24.083 months, respectively. Patients were categorized according to the occurrence of recurrence; 36.4% of patients relapsed within 2 years. In the preliminary analysis, 21 variables were evaluated, including MRI features and histological markers. Significant distinctions were noted in seven characteristics when comparing between the groups, including age (>65 years vs. ≤65 years), AFP (>400 ng/mL vs. ≤400 ng/mL), NLR (≥2.80 vs. <2.80), tumor size (>5 cm vs. ≤5 cm), ITC (incomplete vs. complete), histological grade (well and mediate vs. poor) and microvascular invasion (MVI) (positive vs. negative). Significantly, there were no notable distinctions found in surgical margin width between the recurrence group (median = 2 mm) and the non-recurrence group (median = 2 mm), indicating that the grouping was appropriate. More baseline characteristics are detailed in Table 1.

### 3.2. RFS Pattern in Solitary HCC Patients Undergoing Narrow-Margin Hepatectomy

**Figure 2:**
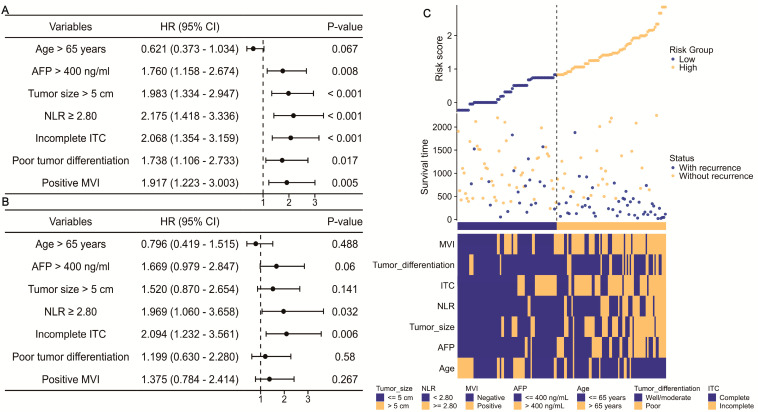
Clinical feature in RFS of solitary HCC patients after narrow-margin hepatectomy. (**A**) Univariate and (**B**) multivariate Cox regression in RFS; (**C**) distribution of the patients’ relapse status with varied clinical characteristics and risk score calculated by Cox model. HR: hazard ratio; CI: confidence interval; AFP: alpha-fetoprotein; NLR: neutrophil-to-lymphocyte ratio; ITC: imaging tumor capsule; MVI: microvascular invasion.

**Figure 3:**
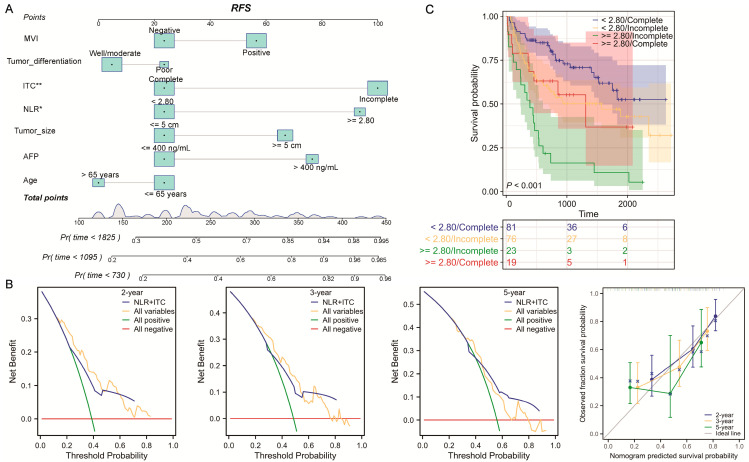
Developed RFS nomogram based on the combination of NLR and ITC. (**A**) RFS nomogram was established based on the combination of NLR and ITC, which were validated via (**B**) DCA and calibration curves in 2-year, 3-year, and 5-year RFS. (**C**) K-M curves of RFS in clinical subgroups stratified by the combination of NLR and ITC. RFS: recurrence-free survival; MVI: microvascular invasion; ITC: imaging tumor capsule; NLR: neutrophil-to-lymphocyte ratio; AFP: alpha-fetoprotein. * *p* < 0.05; ** *p* < 0.01.

Regarding the time of recurrence, univariate Cox regression identified six significant indicators among the seven factors listed above (Figure 2A). In the narrow resection margin group, an NLR ≥ 2.80 [hazard ratio (HR) = 1.969; 95% confidence interval (CI), 1.060–3.658, *p* = 0.032] and incomplete ITC (HR = 2.094; 95% CI, 1.232–3.561, *p* = 0.006) were still independent predictors for RFS (Figure 2B). Using the median risk score of the RFS Cox models as the cutoff value, all patients with a confined resected margin were stratified into low/high-risk groups. As depicted in Figure 2C, patients with a higher risk score had a greater risk of relapses and advanced clinicopathological features, such as a positive MVI, larger tumor size, and incomplete ITC. Furthermore, the RFS nomogram model (C-index = 0.732; 95% CI, 0.700–0.764) based on the Cox models was then constructed (Figure 3A). The DCA and calibration curves, including 2-year, 3-year, and 5-year RFS, were then generated to facilitate the assessment of this nomogram, both of which exhibited excellent validation (Figure 3B). Notably, when focusing on significant variables, combining the NLR and ITC demonstrated a strong predictive value for predicting clinical benefit in RFS, particularly in 5-year RFS. Consequently, we divided patients with solitary HCC who underwent narrow-margin hepatectomy into four categories based on NLR and ITC status. Using the NLR < 2.80 and complete ITC group as a reference, the K-M curves showed a clean and distinct prognostic RFS rate in HCC patient subgroups (*p* < 0.001), with the NLR ≥ 2.80 and incomplete ITC group having the worst RFS (Figure 3C). However, when comparing all other groups to the NLR ≥ 2.80 and complete ITC subgroup, no statistically significant differences were observed, possibly due to the limited sample size. Collectively, integrating preoperative markers, NLR and ITC, could accurately predict RFS for solitary HCC patients after narrow-margin surgery.

### 3.3. Clinical Features of Early Recurrence

These parameters, which demonstrated superior discriminatory ability between groups with and without recurrence, were evaluated for early relapse risk (Table 2). In our cohort, the rate of early recurrence was 36.4%, with a median OS of 21.35 months and RFS of 8.567 months. Among all these variables, no association was found between age > 65 years and the incidence of early recurrence (*p* = 0.084). The most common feature among solitary HCC patients who underwent narrow-margin hepatectomy was an incomplete ITC (69%). Each of the distinctive variables mentioned above was a significant predictor of early recurrence for postoperative patients with a narrow-resected margin, with odds ratio (OR) ranging from 2.395 to 3.513 based on univariate logistic regression (all, *p* < 0.05; Table 3). However, in the multivariate logistic regression, only two predictors remained significant: an AFP > 400 ng/mL (OR = 3.146; 95% CI, 1.304–7.588, *p* = 0.011) and incomplete ITC (OR = 3.220; 95% CI, 1.438–7.209, *p* = 0.004). Notably, our cohort report failed to achieve an NLR ≥ 2.80 as the strikingly independent factor associated with early recurrence in these patients (*p* = 0.053), whereas an AFP > 400 ng/mL was associated with early relapse.

### 3.4. OS Pattern in Solitary HCC Patients Undergoing Narrow-Margin Hepatectomy

**Figure 4:**
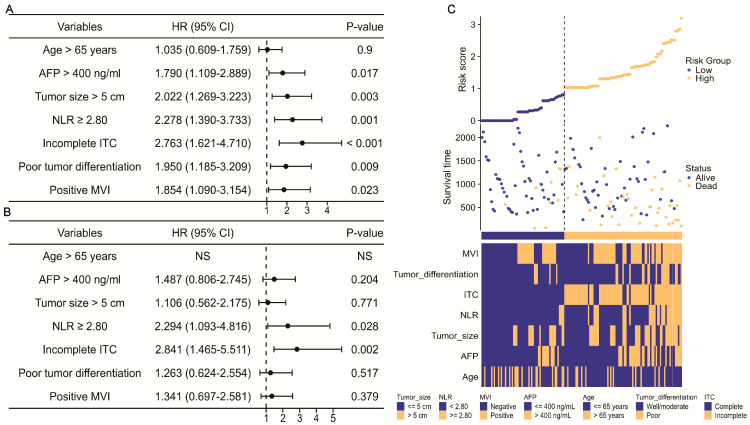
Clinical feature in OS of solitary HCC patients after narrow-margin hepatectomy. (**A**) Univariate and (**B**) multivariate Cox regression in OS. (**C**) Distribution of the patients’ survival status with varied clinical characteristics and risk scores calculated by Cox model. HR: hazard ratio; CI: confidence interval; AFP: alpha-fetoprotein; NLR: neutrophil-to-lymphocyte ratio; ITC: imaging tumor capsule; MVI: microvascular invasion.

**Figure 5:**
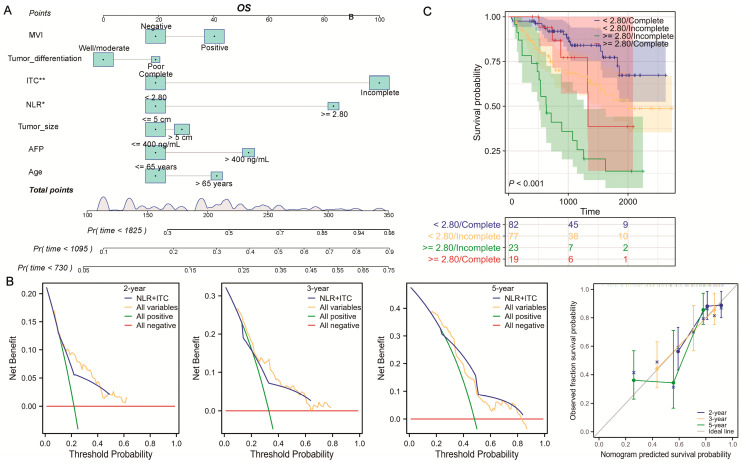
Developed OS nomogram based on the combination of NLR and ITC. (**A**) OS nomograms was established based on the combination of NLR and ITC, which were validated via (**B**) DCA and calibration curves in 2-year, 3-year, and 5-year OS. (**C**) K-M curves of OS in clinical subgroups stratified by the combination of NLR and ITC. OS: overall survival; MVI: microvascular invasion; ITC: imaging tumor capsule; NLR: neutrophil-to-lymphocyte ratio; AFP: alpha-fetoprotein. * *p* < 0.05; ** *p* < 0.01.

Regarding OS in patients with a narrow resection margin, the above seven factors were included in the Cox regression to examine their influences on OS. Consistently, only an NLR ≥ 2.80 (HR = 2.294; 95% CI, 1.093–4.816, *p* = 0.028) and incomplete ITC (HR = 2.841; 95% CI, 1.465–5.511, *p* = 0.002) statistically influenced the OS (Figure 4A,B), showing their powerful predictive ability on OS. Following the findings of the multivariate analysis, there were differences in clinical characteristics between the low- and high-risk groups, with the high-risk group having a less-favorable OS and worse tumor characteristics (Figure 4C). An OS nomogram (C-index = 0.733; 95% CI, 0.692–0.773) was subsequently developed to calculate the patients’ risk scores efficiently and quickly (Figure 5A). The DCA and calibration curves of OS also achieved great performance validation in 2-year, 3-year, and 5-year prognosis (Figure 5B). Intriguingly, similar results as for the RFS were found for the OS. The combination of NLR and ITC also demonstrated its high predictive capability in forecasting clinical benefit in 2-year, 3-year, and 5-year OS. Based on the K-M curves for OS (Figure 5C), patients with an NLR ≥ 2.80 and incomplete ITC had the poorest prognosis, whereas those with an NLR < 2.80 and complete ITC had the best prognosis.

## 4. Discussion

Even with the advent of immunotherapy, hepatectomy remains the most common treatment for HCC patients, as immunotherapy is only effective in a minority of cases [17]. Hence, accurate tumor prognosis following surgical resection remains crucial for implementing effective interventions to prevent relapse and enhance the efficacy of hepatic resection, particularly in HCC with up to 70% recurrence rates [3,4]. Additionally, in most cases, a narrow-resected margin at the radical hepatectomy may be the optimal strategy to preserve more remnant hepatic parenchyma and protect important intrahepatic structures, thereby increasing the likelihood of recovery and enhancing the quality of life for patients, especially those with hepatic dysfunction [18,19]. Therefore, the aim of this study was to evaluate the outcome patterns related to RFS and OS as well as early recurrence in patients treated with radical hepatectomy with a narrow margin, with a particular emphasis on investigating the prognostic impact of the combination-based model of the NLR and ITC.

Chronic inflammation and immune evasion have emerged as prominent hallmarks of cancer [20], especially for HCC characterized as inflammation-associated tumors and ‘cold’ tumors [21,22]. An increase in the NLR indicates that the body is experiencing a systemic inflammatory response and immunosuppression, which leads to tumor progression and a poor prognosis [23]. It is believed that a high NLR is a powerful and independent predictor of HCC survival [24]. This conclusion has been supported by numerous studies with an HR ranging from 1.031 to 4.9 in HCC patients after hepatectomy [25]. In addition, the NLR can be used to stratify HCC patients who are more responsive to therapies dominated by the anti-PD-1/PD-L1 antibody [26]. In our study, an NLR ≥ 2.8 independently impacted the survival outcomes of patients with solitary HCC who underwent narrow-margin hepatectomy, with an HR = 1.969 for RFS and HR = 2.294 for OS. By establishing a score based on the combination of baseline NLR and ITC, patients with a poor prognosis could be identified after resection, allowing for the timely administration of postoperative adjuvant therapy, such as targeted therapy, TACE, or immunotherapy. By increasing the expression of vascular endothelial growth factor (VEGF) and correlating with PD-L1 expression in the tumor core, a high NLR increases the propensity for oncogenesis [27,28].

We also confirmed that an incomplete ITC was associated with a poorer clinical outcome based on MRI characteristics. The precise prognostic impact of tumor capsules remains debatable. Previous research has shown that the long-term survival of HCC is not independently affected by the tumor capsule (*p* = 0.053 in OS), while HCC lacking a fibrous capsule has less histological differentiation and portal vein invasion, indicating superior OS after surgery (*p* = 0.0022) [29]. In contrast, other studies have shown that tumor capsules may inhibit HCC progression and act as an indicator of a favorable prognosis. For instance, HCC patients with capsules show 1-year, 3-year, and 5-year cumulative survival rates of 84%, 70%, and 62%, respectively, while those without capsules show rates of 60%, 40%, and 40%, respectively (*p* = 0.003) [30]. Furthermore, for patients after no-margin hepatectomy, a complete tumor capsule helps to avoid positive resection margins [31]. Consistently, the results of our cohort demonstrated that patients with incomplete ITC undergoing narrow-margin resection had a poor prognosis, with an HR = 2.094 in RFS and HR = 2.841 in OS. In our cohort, HCC with an incomplete ITC tended to exhibit more malignant characteristics, as indicated by AFP levels > 400 ng/mL, an NLR ≥ 2.80, a tumor size > 5 cm, poor tumor differentiation, and positive MVI (Supplementary Table S1). During surgery, an HCC without a complete tumor capsule indicates its infiltrative growth and ambiguous boundary, thereby limiting the extent of surgical resection. This increases the likelihood of residual lesions after hepatectomy and recurrence.

Nomograms based on the NLR and ITC were developed for solitary HCC after narrow-margin hepatectomy. An analysis of samples with and without relapse revealed striking differences in age, AFP, tumor size, NLR, ITC, histological grade, and MVI between the two groups. In contrast, our nomograms for independent factors consisted of preoperative markers based on the NLR and ITC for RFS and OS patterns. AFP, which is commonly utilized in clinical practice [32], was only included in our model for early relapse, which has been reported to reflect a variety of clinical outcomes in hepatic patients whose resection margins are striated. The correlation between these significant results and our clinical relapse criteria is possible. The combination of NLR and ITC has been shown to accurately predict clinical outcomes after narrow-margin hepatectomy in both RFS and OS, which could aid in identifying individuals who may or may not benefit from narrow-margin hepatectomy. Evidently, narrow-margin hepatectomy may not improve the OS for solitary HCC patients with a high NLR and incomplete ITC. Therefore, neoadjuvant therapies and conversion therapies should be considered as alternative treatment options [2].

To the extent of our knowledge, our study is the first to combine NLR- and ITC-based features to establish a prognostic pattern of RFS and OS for patients with solitary HCC after narrow-margin hepatectomy. However, our study encountered several limitations. Due to the fact that our study was retrospective, our nomograms should be prospectively validated for preoperative risk stratification. In addition, given the single center and small sample size, more research involving multicenter and larger samples is required. Since we enrolled a specific population of patients with solitary HCC, the potential generalizability of our model (e.g., patients with multiple nodules) would necessitate calibration testing in multiple populations.

## 5. Conclusions

Solitary hepatic patients undergoing narrow-margin hepatectomy yielded heterogeneous outcomes. Thus, it is crucial to accurately stratify risks in order to optimize patient selection for this surgery. Our nomogram can provide them with precise and individualized risk predictions. Combining preoperative markers primarily based on the NLR and ITC could identify patients who are at a heightened risk of recurrence. Hence, adjuvant therapies (such as radiotherapy, chemotherapy, or immunotherapy) should be recommended. In conclusion, our model could be utilized as a guide for rational clinical treatment and prognosis determination involving the RFS and OS.

## Figures and Tables

**Table 1:** Comparisons of baseline characteristics between hepatic patients undergoing narrow-margin hepatectomy with and without recurrence.

Variables	Total (*N* = 222)	With Recurrence (*n* = 104)	Without Recurrence (*n* = 118)	*p*-Value
Male Gender	184 (82.9)	89 (85.6)	95 (80.5)	0.317
Age > 65 years	53 (23.9)	18 (17.3)	35 (29.7)	0.031
AFP ^a^ > 400 ng/mL	61 (28.9)	35 (35.7)	26 (23)	0.042
ALT, U/L	29.05	28.1	29.25	0.644
AST, U/L	30.4	32.25	29.55	0.067
PT, s	11.95	12	11.9	0.160
INR	1	1	1	0.110
ALBI ≤ −2.63	130 (58.6)	57 (54.8)	73 (61.9)	0.287
Platelet, ×10^9^/L	148	143.5	151	0.713
Child–Pugh grade A	210 (94.6)	97 (93.3)	113 (95.8)	0.412
HBsAg (+)	166 (74.8)	78 (75)	88 (74.6)	0.942
HBeAg (−)	180 (81.1)	84 (80.8)	96 (81.4)	0.911
HCVAb (−)	218 (98.2)	101 (97.1)	117 (99.2)	0.527
NLR ≥ 2.80	44 (19.8)	30 (28.8)	14 (11.9)	0.002
MRI features				
Cirrhosis	106 (47.7)	50 (48.1)	56 (47.5)	0.927
Tumor size > 5 cm	74 (33.3)	43 (41.3)	31 (26.3)	0.017
Incomplete ITC ^a^	100 (49.8)	60 (63.2)	40 (37.7)	<0.001
Histologic markers				
Poor tumor differentiation	39 (17.6)	25 (24)	14 (11.9)	0.017
Histological cirrhosis	143 (64.4)	69 (66.3)	74 (62.7)	0.572
Positive MVI ^a^	82 (49.7)	47 (58)	35 (41.7)	0.036
Ki-67 ^a^ < 20%	64 (32.3)	29 (30.5)	35 (34)	0.604
OS, months	32.283	26.083	37.3	<0.001
RFS ^a^, months	24.083	11.952	37.3	<0.001
Resection margin, mm	2	2	2	0.797

AFP: alpha-fetoprotein; ALT: alanine aminotransferase; AST: aspartate aminotransferase; PT: prothrombin time; INR: international normalized ratio; ALBI: albumin-bilirubin; HBsAg: hepatitis B surface antigen; HBeAg: hepatitis B e antigen; HCVAb: hepatitis C virus antibody; NLR: neutrophil-to-lymphocyte ratio; MRI: magnetic resonance imaging; ITC: imaging tumor capsule; MVI: microvascular invasion; OS: overall survival; RFS: recurrence-free survival; ^a^: some data were missing.

**Table 2:** Comparisons of baseline characteristics between hepatic patients undergoing narrow-margin hepatectomy with early and no early recurrence.

Variables	Total (*N* = 220)	Early Recurrence (*n* = 80)	No Early Recurrence (*n* = 140)	*p*-Value
Age > 65 years	53 (24.1)	14 (17.5)	39 (27.9)	0.084
AFP > 400 ng/mL	61 (29.2)	35 (46.1)	26 (19.5)	<0.001
Tumor size > 5 cm	74 (33.6)	37 (46.2)	37 (26.4)	0.003
NLR ≥ 2.80	44 (20)	25 (31.2)	19 (13.6)	0.002
Incomplete ITC	99 (49.7)	49 (69)	50 (39.1)	<0.001
Poor tumor differentiation	39 (17.7)	21 (26.2)	18 (12.9)	0.012
Positive MVI	81 (49.7)	42 (65.6)	39 (39.4)	0.001
OS, months	32.283	21.35	44	<0.001
RFS, months	24.083	8.567	37.3	<0.001

AFP: alpha-fetoprotein; NLR: neutrophil-to-lymphocyte ratio; ITC: imaging tumor capsule; MVI: microvascular invasion; OS: overall survival; RFS: recurrence-free survival.

**Table 3:** Logistic regression to explore risk factors associated with early recurrence for solitary HCC after narrow-margin resection.

Variables	Univariate Analysis		Multivariate Analysis	
	OR (95% CI)	*p*-Value	OR (95% CI)	*p*-Value
AFP > 400 ng/mL	3.513 (1.886–6.545)	<0.001	3.146 (1.304–7.588)	0.011
Tumor size > 5 cm	2.395 (1.344–4.270)	0.003	1.523 (0.624–3.716)	0.356
NLR ≥ 2.80	2.895 (1.472–5.693)	0.002	2.644 (0.988–7.075)	0.053
Incomplete ITC	3.475 (1.877–6.431)	<0.001	3.220 (1.438–7.209)	0.004
Poor tumor differentiation	2.412 (1.195–4.868)	0.014	1.952 (0.639–5.961)	0.240
Positive MVI	2.937 (1.526–5.652)	0.001	1.687 (0.703–4.049)	0.242

OR: odds ratio; CI: confidence interval; AFP: alpha-fetoprotein; NLR: neutrophil-to-lymphocyte ratio; ITC: imaging tumor capsule; MVI: microvascular invasion.

## Data Availability

Access to the raw data can be obtained by contacting the corresponding author.

## Supplementary Materials

The following supporting information can be downloaded at: https://www.mdpi.com/article/10.3390/jcm13020351/s1, Table S1: Distribution of the significantly prognostic indicators in patients with different ITC.
