# Supplementary material for: Prognostic Value of Combined Neutrophil-to-Lymphocyte Ratio and Imaging Tumor Capsule in Solitary Hepatocellular Carcinoma Patients after Narrow-Margin Hepatectomy
Source: J Clin Med. 2024 Jan 8;13(2):351. doi: 10.3390/jcm13020351 (PMC10816149; doi:10.3390/jcm13020351)
Supplement: Supplementary file 1 [file jcm-13-00351-s001.zip › jcm-2756679-supplementary.pdf]

Supplementary Table S1

Distribution of the significantly prognostic indicators in patients with different ITCs

Variables	Complete ITC (n=101)	Incomplete ITC (n=100)	Total (N=201)
Age > 65 years	31 (30.7)	17 (17)	48 (23.9)
AFP > 400 ng/mL	23 (24)	32 (33.3)	55 (28.6)
NLR ≥ 2.80	19 (18.8)	23 (23)	42 (20.9)
Tumor size > 5 cm	34 (33.7)	35 (35)	69 (34.3)
Poor tumor differentiation	13 (12.9)	23 (23)	36 (17.9)
Positive MVI	33 (44)	41 (53.9)	74 (49)

ITC: imaging tumor capsule; AFP: alpha-fetoprotein; NLR: neutrophil-to-lymphocyte ratio; MVI: microvascular invasion.
